# NLRP3 inflammasome is a key player in human vulvovaginal disease caused by ***Candida albicans***

**DOI:** 10.1038/s41598-017-17649-8

**Published:** 2017-12-19

**Authors:** Elena Roselletti, Stefano Perito, Elena Gabrielli, Antonella Mencacci, Eva Pericolini, Samuele Sabbatini, Antonio Cassone, Anna Vecchiarelli

**Affiliations:** 1 Department of Medicine, University of Perugia, 06132 Sant’Andrea delle Fratte, Perugia, Italy; 2 Department of Diagnostic Medicine, Clinical and Health Public, University of Modena and Reggio Emilia, 41125 Modena, Italy; 3 Polo d’Innovazione di Genomica, Genetica e Biologia, University of Perugia, 06132 Sant’Andrea delle Fratte, Perugia, Italy

## Abstract

The expression of host inflammatory and *Candida albicans* putative virulence factors was studied in women with vulvovaginal candidiasis (VVC; twenty) or colonized by the fungus but asymptomatic (carriers; fifteen) or non-colonized asymptomatic (ten subjects). Overexpression of genes encoding NLRP3 and caspase-1 inflammasome components sharply differentiated VVC patients from asymptomatic colonized or non-colonized women. Inflammasome expression was coupled with neutrophils recruitment in the vagina of VVC women and IL-1β and IL-8 production. Both cytokines were present, though to a lower concentration, also in the vaginal fluid of colonized and non-colonized women. Secretory aspartyl proteinases (*SAPs*) and hyphae associated genes *HWP1* and *ECE1* were upregulated in VVC but with some differences among infected women. The most overexpressed *SAP* gene was *SAP2*, that correlated with neutrophils accumulation. Our data provide clinical evidence that the intracytoplasmic activation of NLRP3 inflammasome complex plays a critical, pathogenesis-relevant role in human VVC.

## Introduction

*Candida albicans* (*C*. *albicans*) is a human commensal fungus which colonizes mucosal surfaces, including the gastrointestinal and vaginal tract. It is also one of the most common fungal pathogens responsible for both superficial as well as life-threatening, deep-seated infections in immune-compromised or otherwise debilitated host. The most common superficial infection caused by this fungus is vulvovaginal candidiasis (VVC), that acutely affects at least once in their life around 75% of women of childbearing age and a relevant portion of them (6 to 8%) in chronic, recurrent form (RVVC)^1,2^. The microbiological and immune-pathological factors determining VVC and RVVC have long been studied, particularly using rodent models^3,4^. Recent data from murine models suggest that vaginal disease caused by *C*. *albican*s is critically determined by activation of microbial and/or host factors leading to vaginal inflammation with dominance of neutrophils (PMN) which are unable to resolve the fungal infection^5,6^. However, the evidence about fungus persistence and mechanisms of inflammatory responses as the pathogenic determinant of human VVC and RVVC is limited^7^. In addition, it has remained unclear whether rodent models are a proxy of human disease^4^. Little is also known about the fungal factors which cause the loss of the typical immune tolerance exerted by the vaginal environment and concur to trigger local inflammation. Potential candidates are one or more of the numerous virulence attributes of *C*. *albicans* that include enzymes, adhesins and growth under hyphal form which, on one side, appear to deceive and, on the other side, to overstimulate the host response^8^.

Among the above virulence attributes, a special consideration has been reserved to the secretory aspartyl proteinases (Saps) proteins family. These are encoded by at least ten genes (*SAP1-10)* encoding members of Sap1-3, Sap4-6, Sap7, Sap8 and Sap9-10 subfamilies^9^. Previous studies in experimental rodent models have shown that *SAPs* are indeed strongly expressed during both rat and mouse vaginal infections, and various levels of expression of the different *SAPs* have also been found in human VVC^10^. More recently, *SAPs* expression and Saps activity in recruiting vaginal neutrophils and accompanying production of activating soluble factors have been shown to play a role in murine vaginal inflammation, though different Saps have been implicated as main mechanistic pro-inflammatory factors in different studies^3,6,8,11^. Finally, other investigators have identified hyphae-associated *C*. *albicans* proteins with a possible role in infection and inflammation^12,13^. Nonetheless, the role, if any, played by the above fungal factors in human disease remains uncertain or simply unexplored. For these reasons, we have here addressed the expression of critical components of the inflammasome machinery and inflammatory neutrophil-recruiting and activating cytokines in the vaginal secretion of women with clinically established VVC. This has been coupled with the expression of fungal factors, including *SAPs*, in the same clinical materials. The vaginal material taken from asymptomatic women harboring *C*. *albicans* in their vagina served as control.

## Results

### Infection *versus* Carriage

**Figure 1:**
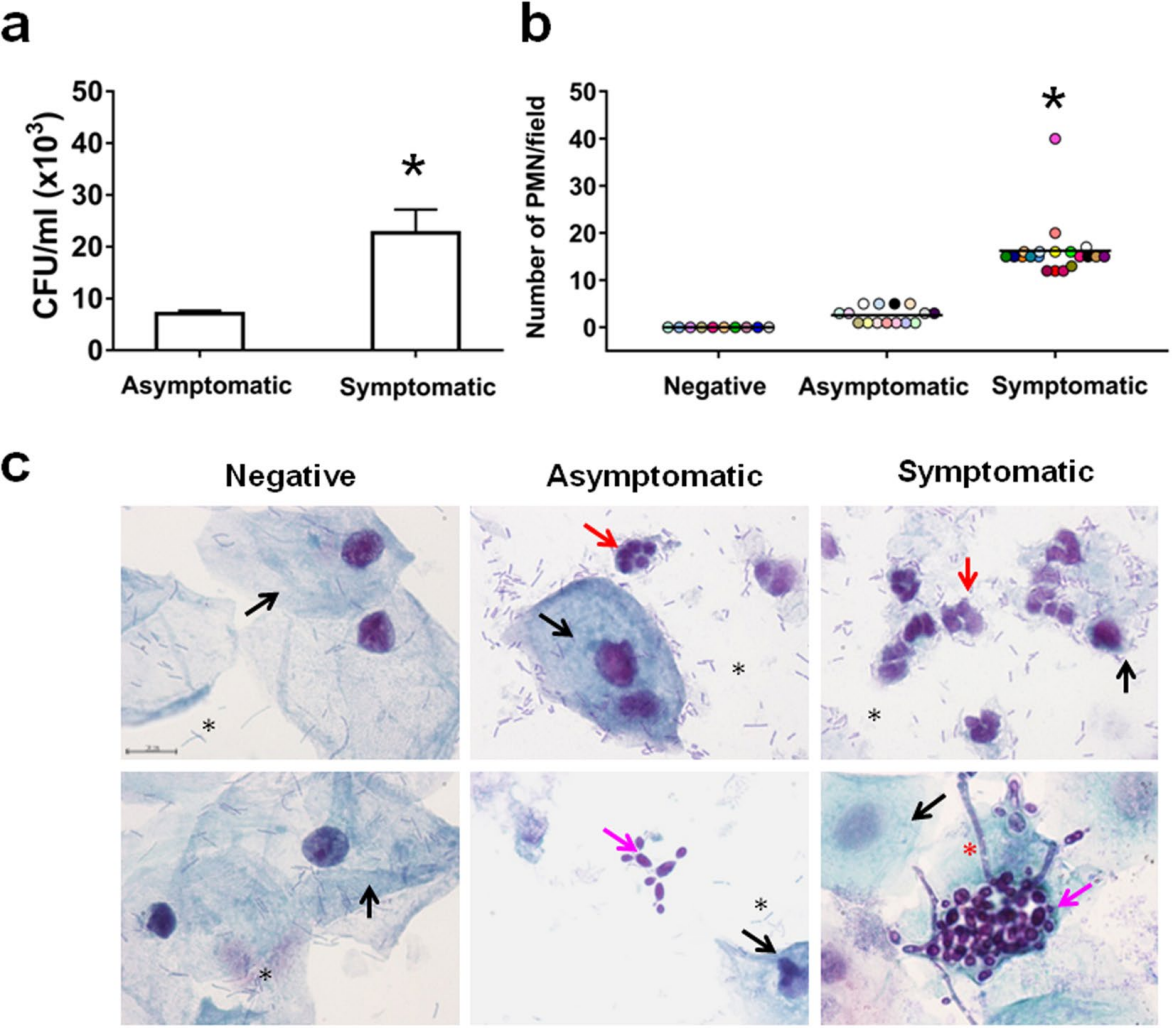
Determination of fungal burden and PMN infiltration in vaginal samples. The CFU in vaginal samples of asymptomatic (n = 5) and symptomatic (n = 5) women were evaluated and the statistical significance was determined with Student’s t test. Data are expressed as mean ± SEM. *p = 0.016 symptomatic *vs* asymptomatic women (**a**). Vaginal samples of negative (n = 10, identified by dots with different colors), asymptomatic (n = 15, identified by dots with different colors) and symptomatic (n = 20, identified by dots with different colors) women were examined under light microscope to evaluate the PMN recruitment by morphology. The statistical significance was determined with Mann Whitney U test. *p = 3.07 × 10^−10^ symptomatic *vs* asymptomatic women (**b**). Vaginal samples of negative (n = 10), asymptomatic (n = 15) and symptomatic (n = 20) women were microscopically examined to evaluate the presence of epithelial cells (**→**), PMN (**→**), lactobacilli (*) and *C*. *albicans* in yeast (**→**) or hyphal form (*) (original magnification x1000, bar = 10 μm). Representative images of each kind of vaginal samples (upper and lower panels) shown are from three different women (**c**).

Vaginal samples were taken from women who fulfilled the VVC case definition (n = 20) or were asymptomatic carriers (n = 15). Vaginal samples from 10 healthy asymptomatic and fungus-negative subjects also entered the study for control purposes. All samples were examined for the presence of inflammatory and fungal cells, and scored as described in Methods and Table 1. Ten randomly selected vaginal samples, five from asymptomatic carriers and five from VVC cases were examined for fungal burden. As shown in Fig. 1a, the fungus burden was around three times higher in the selected symptomatic than asymptomatic women. Moreover, the vaginal samples of the former subjects contained a consistent number of PMN (16.2 ± 1.3 cells per field, mean ± SEM; score 2), while PMN were scarcely or not detectable (2.6 ± 0.4 cells per field, mean ± SEM; score 1) in *Candida*-carriers, asymptomatic subjects. No PMN were found in the vaginal samples of the ten asymptomatic, non *Candida*-carriers (Fig. 1b). Visual inspection of fungal morphology showed that the vaginal samples of *Candida*-carriers contained only yeast and some pseudo-mycelial forms, whereas the vaginal samples of symptomatic, *C*. *albicans*-infected women had preponderant yeast cells but also variable quantities of pseudo-mycelial and hyphal cells roughly in the average proportion of 2:1 yeasts *vs* pseudyphal-hyphal forms (Fig. 1c).

**Table 1:** Characteristics of women enrolled in the study. PMN scores from 0 to 2 and clinical signs and symptoms from vaginal samples obtained from women enrolled in the study are shown.

	Negative for *C*. *albicans*	Positive for *C*. *albicans* and asymptomatic	Positive for *C*. *albicans* and symptomatic
Vaginal samples	10	15	20
PMN scores	0	1	2
Clinical signs and symptoms	no	no	vaginal discharge, itching, burning, dyspareunia

### Vaginal inflammation

**Figure 2:**
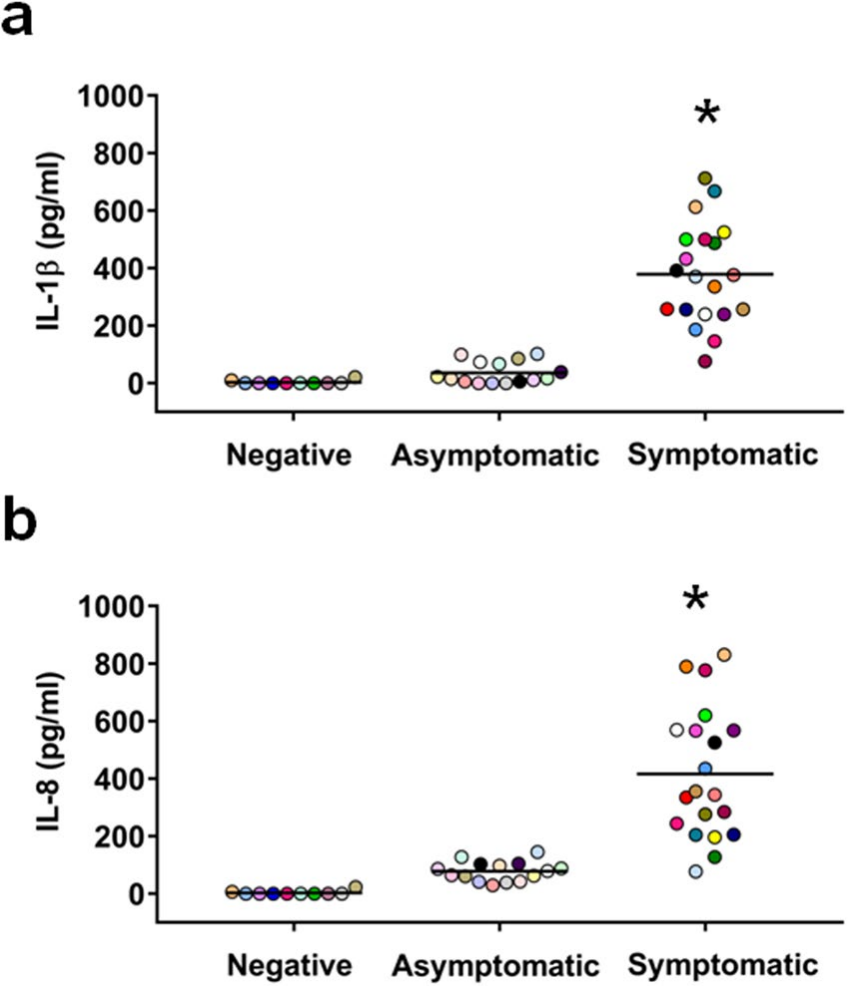
IL-1β and IL-8 production in vaginal samples. The supernatants of vaginal samples of negative (n = 10, identified by dots with different colors), asymptomatic (n = 15, identified by dots with different colors) and symptomatic (n = 20, identified by dots with different colors) women were tested for IL-1β (**a**) and IL-8 (**b**) production by specific ELISA assays. The results are from triplicates samples of each subjects and the statistical significance was determined with Mann Whitney U test. *p = 2.1 × 10^−9^ symptomatic *vs* asymptomatic women for IL-1β. *p = 4.2 × 10^−8^ symptomatic *vs* asymptomatic women for IL-8.

The distinctive presence of abundant PMN in the vaginal samples of subjects with VVC suggested for an active vaginal inflammatory process. To corroborate this hypothesis, we first explored whether pro-inflammatory cytokines, such as IL-8 and IL-1β, which are critically involved in PMN recruitment and activation, were commonly present in the vaginal samples of symptomatic women. Both cytokines were indeed detected in the vaginal fluid of all women. However, their concentration was remarkably higher in symptomatic respect to asymptomatic subjects (mean concentrations of symptomatic and asymptomatic were 378.6 ± 38.2 *vs* 36.3 ± 9.6, *p* = 2.1 × 10^−9^ for IL-1β and 416.7 ± 49.5 *vs* 78.3 ± 8.4, *p* = 4.2 × 10^−8^ for IL-8, respectively) (Fig. 2). Very low to undetectable levels of IL-1β and IL-8 were found in the vaginal samples of the ten asymptomatic, non *Candida*-carriers subjects (Fig. 2) where no PMN were found (Fig. 1).

**Figure 3:**
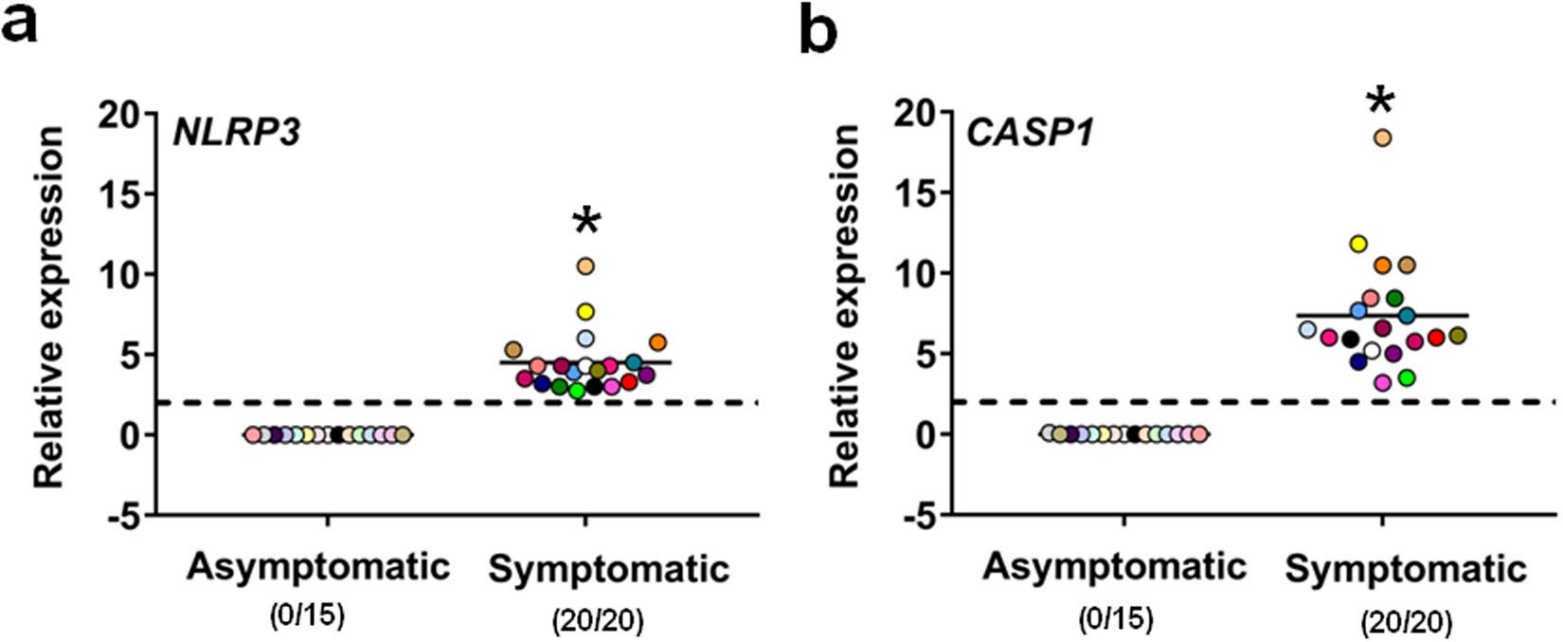
Quantitative analysis of *NLRP3* and *CASP1* gene expression. Vaginal samples of asymptomatic (n = 15, identified by dots with different colors) and symptomatic (n = 20, identified by dots with different colors) women were centrifuged at 3000 rpm for 10 min, then cellular fractions were lysed and total RNA was extracted and retro-transcribed in cDNA. The expression levels of *NLRP3* (**a**) and *CASP1* (**b**) genes in asymptomatic and symptomatic women were calculated by comparative Ct method (2^−∆Ct^ formula) after normalization with *GADPH* gene. The results are from triplicates samples of each subject and the statistical significance was determined with Mann Whitney U test. The dashed line denotes the cutoff at which the overexpression of *NLRP3* and *CASP1* genes was defined as >2 times the value of *GADPH* expression and the number of women expressing genes is indicated in the brackets. *p = 3 × 10^−10^ symptomatic *vs* asymptomatic women for *NLRP3* and *CASP1*.

The above cytokines are strongly involved in the inflammatory response which mostly relies upon activation of NLRP3, a critical component of inflammasome complex that initiates the inflammatory response through the activation of caspase-1. Activated caspase-1 cleaves the pro-IL-1β to active secreted IL-1β, a key inflammatory mediator driving the host response to infection^14^. We therefore verified the expression of NLRP3 and caspase-1 in our vaginal samples. As shown in Fig. 3, both *NLRP3* and *CASP1* were found to be expressed in the vaginal samples of all symptomatic subjects, to a different degree depending on the subject tested, but not in those of asymptomatic, colonized women (Fig. 3a and b). No expression of the above inflammasome components was either found in asymptomatic, non-colonized women (data not shown).

### Secretory aspartyl proteinases

**Figure 4:**
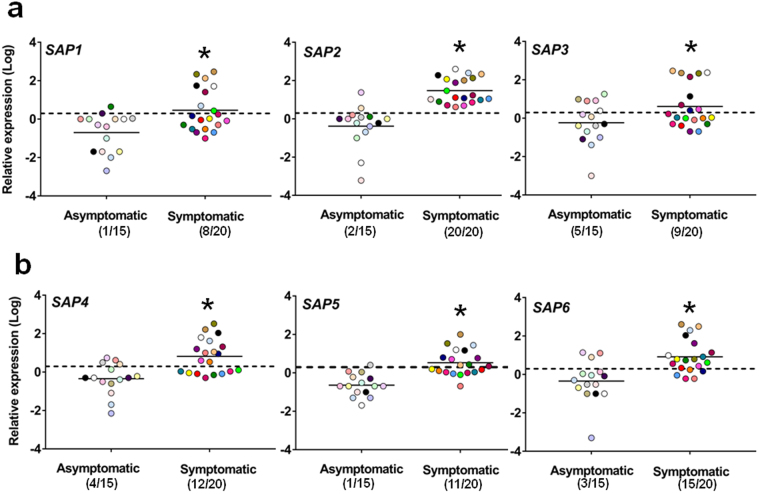
Quantitative analysis of *SAP1-6* gene expression. Vaginal samples of asymptomatic (n = 15, identified by dots with different colors) and symptomatic (n = 20, identified by dots with different colors) women were centrifuged at 3000 rpm for 10 min, then cellular fractions were lysed and total RNA was extracted and retro-transcribed in cDNA. The expression levels of *SAP1*, *SAP2*, *SAP3* (**a**) and *SAP4*, *SAP5*, *SAP*6 (**b**) genes in asymptomatic and symptomatic women were calculated by comparative Ct method (2^−∆Ct^ formula) after normalization with *ACT1* gene. The results are from triplicates samples of each subject and the statistical significance was determined with Mann Whitney U test. The dashed line denotes the cutoff at which the overexpression of *SAP1*-*6* genes was defined as >2 times the value of *ACT1* expression and the number of women expressing genes is indicated in the brackets. *p = 0.01168 symptomatic *vs* asymptomatic women for *SAP1*; *p = 5.8 × 10^−8^ symptomatic *vs* asymptomatic women for *SAP2*; *p = 0.04212 symptomatic *vs* asymptomatic women for *SAP3*. *p = 0.00014 symptomatic *vs* asymptomatic women for *SAP4*; *p = 3.4 × 10^−6^ symptomatic *vs* asymptomatic women for *SAP5*; *p = 0.00029 symptomatic *vs* asymptomatic women for *SAP6*.

**Figure 5:**
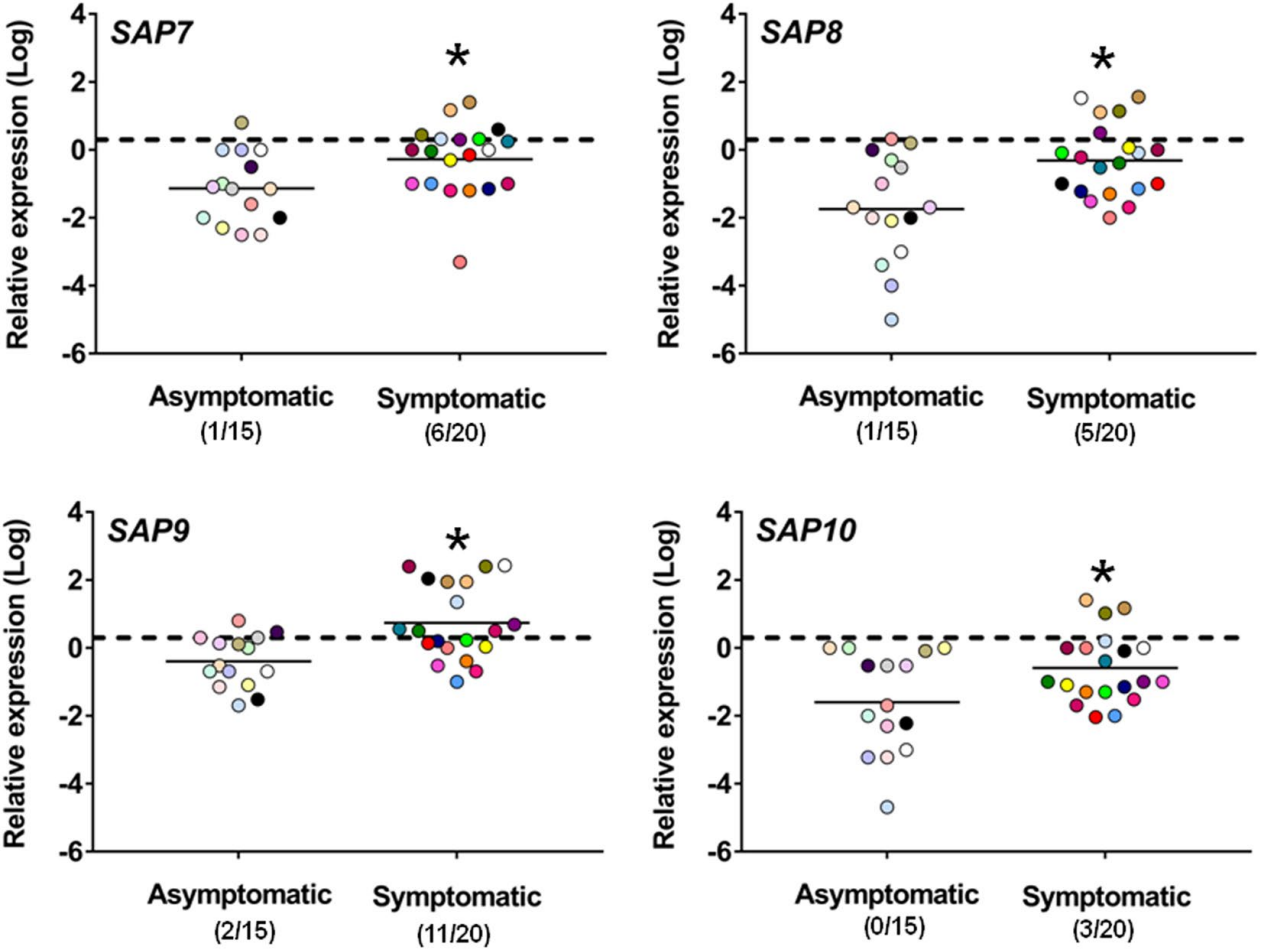
Quantitative analysis of *SAP7-10* gene expression. Vaginal samples of asymptomatic (n = 15, identified by dots with different colors) and symptomatic (n = 20, identified by dots with different colors) women were centrifuged at 3000 rpm for 10 min, then cellular fractions were lysed and total RNA was extracted and retro-transcribed in cDNA. The expression levels of *SAP7*, *SAP8*, *SAP9* and *SAP10* genes in asymptomatic and symptomatic women were calculated by comparative Ct method (2^−∆Ct^ formula) after normalization with *ACT1* gene. The results are from triplicates samples of each subject and the statistical significance was determined with Mann Whitney U test. The dashed line denotes the cutoff at which the overexpression of *SAP7-10* genes was defined as >2 times the value of *ACT1* expression and the number of women expressing genes is indicated in the brackets. *p = 0.00961 symptomatic *vs* asymptomatic women for *SAP7*; *p = 0.00457 symptomatic *vs* asymptomatic women for *SAP8*; *p = 0.00165 symptomatic *vs* asymptomatic women for *SAP9*; *p = 0.02851 symptomatic *vs* asymptomatic women for *SAP10*.

It has long been suggested that one or more proteins of ten member of Saps family of *C*. *albicans* exerted a role in the immune-pathogenesis of VVC and RVVC^6,10,15–19^. More recently, some Saps, particularly Sap2, Sap5 and Sap6 have been reported as inducers of inflammation in murine models, suggesting that they could mediate the pathogenesis of human vaginal candidiasis^5,6,8^. Therefore *SAP1-10* expression was evaluated in all vaginal samples of symptomatic and asymptomatic subjects. No *SAP1-10* expression was detected in vaginal samples which were negative for *C*. *albicans* (n = 10). Moreover, *SAP1-10* were scarcely overexpressed and generally only in few of the asymptomatic *Candida*-carriers (Figs 4 and 5). In contrast, expression of each *SAP* was upregulated in at least some of symptomatic women (Figs 4 and 5). Overall, of the *SAPs* most studied in the immune-pathogenesis and inflammation context, *SAP2* was upregulated in all patients, and to high level, in some of them (Fig. 4a). *SAP1* and *SAP3* were expressed in roughly one half of symptomatic women (Fig. 4a). The hypha-associated *SAP* such as *SAP6* was overexpressed in a major part of symptomatic women; *SAP4* and *SAP5* were moderately overexpressed in roughly one half of the subjects (Fig. 4b). Of note, the cell wall *SAP9* and *SAP10*, called yapsins^20^, and also *SAP7* and *SAP8* resulted overexpressed in symptomatic subjects (Fig. 5).

### Hyphae-associated virulence genes

**Figure 6:**
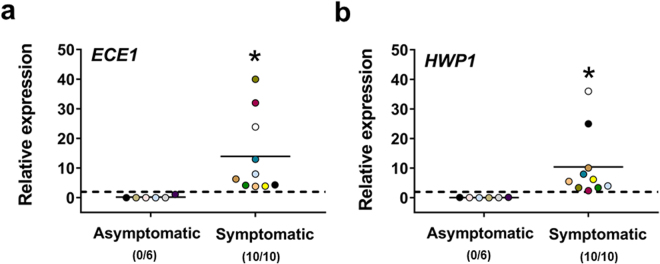
Quantitative analysis of *ECE1* and *HWP1* gene expression. Vaginal samples of asymptomatic (n = 6, identified by dots with different colors) and symptomatic (n = 10, identified by dots with different colors) women were centrifuged at 3000 rpm for 10 min, then cellular fractions were lysed and total RNA was extracted and retro-transcribed in cDNA. The expression levels of *ECE1* (**a**) and *HWP1* (**b**) genes in asymptomatic and symptomatic women were calculated by comparative Ct method (2^−∆Ct^ formula) after normalization with *ACT1* gene. The results are from triplicates samples of each subject and the statistical significance was determined with Mann Whitney U test. The dashed line denotes the cutoff at which the overexpression of *ECE1* and *HWP1* genes was defined as >2 times the value of *ACT1* expression and the number of women expressing genes is indicated in the brackets. *p = 0.00012 symptomatic *vs* asymptomatic women for *ECE1* and *HWP1*.

Hyphal cells of *C*. *albicans* appeared to be only a relatively minor component of the fungal forms present in the vaginal samples of women with VVC (see Fig. 1), and a typical hyphae-associated *SAP* gene, *SAP5*, was not the one mostly expressed in our patient cohort, while *SAP6* was expressed in about 75% of symptomatic women (see Fig. 4b). Nonetheless, we were interested in the expression of hyphae-associated genes for the role usually assigned to the hyphal development in *C*. *albicans* pathogenesis and particularly in vaginal infection and inflammation^12,21^. Therefore, we examined the expression of two pathogenesis-relevant hyphae associated genes such as *ECE1* and *HWP1* in our vaginal samples. In particular, *ECE1* was selected because it encoded a precursor of the recently identified candidalysin toxin^13^. As shown in Fig. 6 the expression of both the above *C*. *albicans* genes was negligible in the asymptomatic *Candida*-carriers. In contrast, both *ECE1* and *HWP1* were expressed to substantial levels in all vaginal samples of tested symptomatic women.

## Discussion

VVC is a common infection in most of otherwise healthy women of childbearing-age, and in a quite remarkable proportion of them it develops as highly distressing chronic, recurrent forms (RVVC)^1,2^.

These are usually caused by *C*. *albicans* but can occasionally be caused by other non *albicans* species such as *C*. *krusei*, *C*. *tropicalis*, *C*. *glabrata* ^22^. *C*. *albicans* is a common type of fungus, often found in small amounts in the vagina, mouth, digestive tract, and on the skin without causing infection or symptoms, but can switch from the commensal state to a pathogenic one. In mouse models, experimental vaginal infection by *C. albicans* results in a strong inflammatory response with a marked leukocyte infiltrate essentially consisting of polymorphonuclear cells (neutrophils) and activation of caspase-1/inflammasome components^5,6^. These studies have also shown that vaginally recruited neutrophils are unable to eliminate or substantially restrict the fungus growth in the vagina, likely not due to the loss of intrinsic candidacidal capacity but to the presence of *Candida*- or host-derived inhibitors^11,23^. Eventually the constant presence of high numbers of fungal cells, the release of inflammation-inducing constituents, such as, for instance, Sap^6^ and candidalysin^13,24^, together with the inflammatory cytokines actually released by the leukocytes and epithelial cells cause an acute inflammatory state. However, the extent to which mouse and other animal models of vaginal candidiasis are faithful representatives of human infection is unclear^4^. Unlike *C*. *albicans*, *C*. *glabrata*, unable to make hyphae and produce Sap *in vitro* ^9^, did not elicit an inflammatory immunopathogenic response in a murine model of vaginitis^25^.

In this paper we addressed vaginal inflammation in women with VVC in comparison with asymptomatic *C*. *albicans* vaginal carriers and non-colonized, healthy women. Inflammation was defined by the presence of neutrophils, pro-inflammatory cytokines and expression of inflammasome components. The NLRP3 inflammasome is an intracellular receptor complex that plays a key role in most inflammatory diseases via activation of caspase-1 that leads to cleavage of proIL-1β to the biologically active IL-1β^26^. It has been recently reported that it could be also activated by other non *albicans* species such as *C*. *parapsilosis* ^27^.

Polymorphisms of, and presence of variable number tandem repeats in, the *NLRP3* gene have been variably associated to VVC or RVVC^28,29^ but to our knowledge, in none of the previous studies the expression of NLRP3 inflammasome and caspase-1 components in women with VVC has been detected and quantified. We here report a rather sharp difference between asymptomatic carriers and symptomatic infected women in that only the latter showed evidence for consistent *NLRP3* inflammasome and *CASP1* gene overexpression in their vaginal cells. This finding rather than the fungal burden or even the presence of cytokines themselves appears to be a true landmark of VVC. In fact, the difference between the CFUs detected in the vagina of VVC or asymptomatic carriers was relatively low (only roughly three times) and cytokines such as IL-1β and IL-8 were also consistently present, though to a lower concentration, also in the vaginal fluid of asymptomatic vaginal carriers. Low levels of IL-1β and IL-8 were also present in the vaginal fluid of healthy, non-colonized women who, as the asymptomatic carriers, had no expression at all of *CASP1* and *NLRP3* inflammasome. These data provide evidence that *NLRP3* inflammasome expression is also a consistent marker of VVC in humans as it appears to be in two different murine models^5,6^. It is unclear whether vaginal epithelial cells are those uniquely expressing NLRP3 or the neutrophils could contribute to that expression, also in view of their resistance to apoptosis in an inflamed, cytokine-rich medium^30^. Further studies will address this interesting aspect.

Our recent investigations have shown that Sap2 and Sap6 are likely involved in inducing inflammatory response by human monocytes, macrophages, and dendritic cells *in vitro*, through activation of NLRP3 inflammasome and induction of different caspases^31,32^. In a more recent paper, by using a murine model^6^ we demonstrated that several Saps, particularly Sap2, cause a sort of aseptic inflammatory response in the mouse vagina in the absence of fungal cells. Since anti-Sap2 antibodies inhibited vaginal inflammation caused by *C*. *albicans*, it was proposed that one or more Saps could be the inflammation mediator of fungal disease. For the above reasons, we further studied the expression of the ten genes coding for Saps family in women with VVC and in fungus carriers. While these latter had very faint expression of some of the above genes, a variable but in some VVC women elevated overexpression of some of *SAPs* genes was detected. In particular, *SAP2* expression was variably though consistently upregulated in the vaginal samples of all VVC patients. Other most frequently upregulated *SAPs* were *SAP6*, *SAP4*, *SAP5* and *SAP9* in this ranking order. *SAP6* was overexpressed in 15 samples over 20 tested, *SAP4* in 12 samples, *SAP5* and *SAP9* in 11 samples. Comparison with quantitative *SAPs* genes expression to other studies is impossible because of the differences in women recruitment, different case definition and times of VVC disease. Nonetheless, our data are qualitatively similar to those reported by Naglik and collaborators^10^ with the possible exception of *SAP5* and *SAP10* genes expression that was detected only in some of VVC patients.

The relatively infrequent overexpression of the *SAP5* gene, that is known to be hyphae associated, could be due to the relatively minor abundance of hyphal cells in the vaginal samples of our VVC patients. The fungal morphology of our samples showed a consistent presence of yeast with a lower proportion of some hyphae and pseudohyphae in symptomatic patients, with never massive presence of hyphae. This could be due to the exclusion of pregnant women from our cohort. In other studies (data not shown here) we noted that hyphae were much more consistently present in vaginal samples from pregnant VVC and RVVC subjects (unpublished data). In agreement with data by others^10^, no or limited expression of any *SAPs* gene was detected in non-VVC asymptomatic patients.

Hyphal growth of *C*. *albicans* is recurrently advocated to play an important role in mucosal infection by this fungus^12,21^. Although pseudohyphal and hyphal cells were less abundant than yeast cells in the vaginal samples of our symptomatic patients, we were interested in measuring the expression of putative hyphae-associated genes in our patients. To this purpose, among all the hyphae-associated genes we selected two genes, the *HWP1* which codes for a fungal cell wall protein, required for hyphal development^33^, and *ECE1* which is highly expressed by hyphae during infection of epithelial cells^13,34^. Hwp1 and Ece1 displayed a key role in two phases of *C*. *albicans* pathogenesis: adhesion and tissue damage. During *C*. *albicans* mucosal infection the fungal interaction to epithelial cells promoted the formation of hyphae which strengthen fungal adhesion. This link was mediated by adhesins expressed on hyphae such as Hwp1^35^.

We found that both *HWP1* and *ECE1* were overexpressed in symptomatic patients, but not or very low so in asymptomatic carriers. This is in apparent contrast with previous studies reporting rather similar expression of *HWP1* gene in both symptomatic and in asymptomatic women^12^. *ECE1* is one of hyphal genes in *C*. *albicans* which has been identified in 1990^36^. This gene codes for the recently discovered toxin, named candidalysin^13^. The candidalysin is reported to directly damage host epithelial membranes, to trigger a danger response signaling pathway and activate epithelial immunity^13^. We found upregulated expression of *ECE1* in all VVC patients tested, while not in asymptomatic carriers. To our knowledge, this is the first demonstration that *ECE1* is overexpressed during human vaginal candidiasis.

This study has some limitations concerning the non elevated number of patients examined, the exclusion of non-*C*. *albicans* infected VVC subjects and the lack of quantitative distinction of the different *C*. *albicans* forms of growth in the vaginal samples of VVC subjects. Despite this, the data overall suggest that NLRP3 inflammasome expression and the consequent pro-inflammatory cytokine cascade play a central role in VVC. They also indirectly confirm that the overexpression of one or more *SAPs* and possibly other fungal components, including the recently discovered candidalysin peptide from the Ece1 protein^13^, rather than the fungal burden per se, could play an important role in triggering the inflammatory cascade. These data strengthen some mechanistic similarity between VVC and vaginal inflammation in mouse models^37^. However, if this similarity is also applicable to the chronic, recurrent forms of vaginal candidiasis remains an open question.

## Methods

### Subjects

Fortyfive non-pregnant, non-diabetic women, 19 to 53 years old, attending the microbiological diagnostic service of the University Hospital Santa Maria della Misericordia, Perugia (Italy) over the period from February 2016 to April 2017 were consecutively enrolled in this study. This cohort included *C*. *albicans*-colonized symptomatic (n = 20), *C*. *albicans*-colonized asymptomatic (n = 15) and *C*. *albicans*-non colonized asymptomatic (n = 10) women. Prior to enrollment, each subject answered a questionnaire indicating their health status and current symptoms of vaginal disease. A case of VVC due to *C*. *albicans* was defined by the isolation of *C*. *albicans* from the vaginal sample and the presence of at least two of the following signs and symptoms: vaginal discharge, itching, burning and dyspareunia. None of the recruited women had RVVC as indicated by the absence of documented or woman-reported, repeated VVC episodes per year. All women signed an informed consent in accordance with the Declaration of Helsinki. Local Ethical Committee CEAS (Comitato Etico delle Aziende Sanitarie, Umbria, Italy) approval was received for the whole study (VAG1 n. 2652/15). All methods were performed in accordance with the relevant guidelines and regulations.

### Samples collection

A vaginal swab was taken from each participant and soaked in 1 ml of saline. After sampling, the vaginal swab was plated on CHROMagar^TM^ Candida (VWR International p.b.i., Milan, Italy) at 37 °C for 48 h to evaluate the vaginal colonization by *C*. *albicans*. The presence of *C*. *albicans* was also confirmed by MALDI-TOF test (Biomérieux S.A. France). The vaginal fluid was serially diluted and plated on CHROMagar^TM^ Candida (VWR International p.b.i.) to quantify the vaginal fungal burden^7^. Subsequently, the vaginal fluid was centrifuged at 3000 rpm for 10 min, then cellular fraction was used for gene expression analysis and supernatant for cytokine production.

### Neutrophil infiltration analysis

Vaginal samples were examined under light microscope (Olimpus, Milan, Italy) to evaluate the presence of *C*. *albicans* in yeast or hyphal form and neutrophils (PMN) by their morphology after staining with Papanicolaou technique^7^. The number of PMN was counted in four fields at x400 magnification and expressed as average number of PMN/field. The PMN score was graded on a scale from 0 to 2: 0, 0 PMN/field; 1, 1 to 10 PMN/field; 2, 11 to 40 PMN/field.

The *C*. *albicans*-colonized symptomatic women showed at least two of the following symptoms: vaginal discharge, itching, burning, dyspareunia, associated with PMN infiltration score 2 and *C*. *albicans* isolation from the vaginal sample.

The *C*. *albicans*-colonized asymptomatic women showed *C*. *albicans* isolation from the vaginal sample, PMN score 1 but not clinical symptoms.

The *C*. *albicans*-non colonized women did not show *C*. *albicans* isolation from the vaginal sample, PMN infiltration (score 0) and clinical symptoms.

### Cytokine Production

The supernatants of vaginal samples were collected and tested for IL-1β and IL-8 production by specific ELISA assays (all from eBioscence, San Diego, CA). Cytokine titers were calculated relative to standard curves.

### Quantitative analysis of *SAP1-10*, *ECE1*, *HWP1*, *NLRP3* and *CASP1* gene expression in vaginal samples

The cellular fractions of vaginal samples were lysed using Trizol (Life Technologies, Monza, Italy). Total RNA was extracted and retro-transcribed by using the Moloney murine leukemia virus reverse transcriptase reaction (M-MLV RT), as described in the manufacturer’s instructions. cDNA concentration was determined using a spectrophotometer. Human *GADPH*, *NLRP3*, *CASPASE1* (*CASP1*) and *C*. *albicans ACT1*, *SAP1*, *SAP2*, *SAP3*, *SAP4*, *SAP5*, *SAP6*, *SAP7*, *SAP8*, *SAP9*, *SAP10*, *ECE1* and *HWP1* gene expression was detected by using primers reported elsewhere and showing similar capacity and efficiency in detecting expression of the above genes^10,12,13,38,39^. Real-time PCR (quantitative PCR) was performed in 96-well PCR plates (Thermo Scientific, Waltham, MA USA) using SYBR green (BioRad, Milan, Italy). For real-time PCR reaction 200 ng of cDNA was used. All samples were measured in triplicates. The expression levels of *SAP1-10*, *ECE1*, *HWP1*, *NLRP3* and *CASP1* genes in asymptomatic and symptomatic women were calculated by comparative Ct method (2^−∆Ct^ formula) after normalization with *ACT1* for *C*. *albicans* genes and *GADPH* for human genes^40–42^. Amplification conditions were the same used for *ACT1*, *SAP1-10*, *ECE1*, *HWP1*, *NLRP3*, *CASP1* and *GADPH* genes: 3 min at 95 °C, 40 cycles of 10 sec at 95 °C and 30 sec at primer specific temperature. The experiments were performed using Applied Biosystems 7300 (Thermo Scientific). Overexpression of relevant genes was defined as >2 times the value of housekeeping gene expression.

### Statistical Analysis

Results reported in the dot plot graphs were from triplicate samples of all vaginal fluids; results reported in the bar graphs were the mean ± SEM from triplicates samples of 5 asymptomatic or symptomatic vaginal fluids.

Quantitative variables were tested for normal distribution by using SigmaPlot 12.5 program. For CFU count the quantitative variables were compared by means of Student’s two-tailed *t* test. For other determinations the quantitative variables were compared by Mann Whitney U test.

Values of *p* < 0.05 were considered significant.
